# The Prediction of Response to Galantamine Treatment in Patients with Mild to Moderate Alzheimer’s Disease

**DOI:** 10.2174/15672050113106660167

**Published:** 2014-02

**Authors:** Takashi Ohnishi, Yojiro Sakiyama, Yuichi Okuri, Yuji Kimura, Nami Sugiyama, Takayuki Saito, Masayoshi Takahashi, Takumi Kobayashi

**Affiliations:** 1 Scientific Affairs Division, CNS Science Department, Janssen Pharmaceutical K.K., Tokyo, Japan; 2 Research and Development Division, Department of Neuroscience, Janssen Pharmaceutical K.K., Tokyo, Japan; 3 Research and Development Division, Department of Clinical Science Initiatives, Janssen Pharmaceutical K.K., Tokyo, Japan

**Keywords:** Acetylcholinesterase inhibitor, Alzheimer's disease, Alzheimer’s disease assessment scale-cognitive subscale (ADAS-cog), galantamine, pharmacotherapy, responders.

## Abstract

The prediction of efficacy in long-term treatment of acetylcholinesterase inhibitors (AChEIs) is a major clinical issue, although no consistently strong predictive factors have emerged thus far. The present analyses aimed to identify factors for predicting long-term outcome of galantamine treatment. Analyses were conducted with data from a 24 weeks randomized, double-blind, placebo controlled trial to evaluate the efficacy and the safety of galantamine in the treatment of 303 patients with mild to moderate AD. Patients were divided into responders (4 or more point improvement of ADAS-cog scores at 24 weeks of treatment) and non-responders. We explored whether patients’ background (e.g. sex, age, and duration of disease) and scores of cognitive scales at early stage, are relevant to the long-term response to AChEIs. Predictive values were estimated by the logistic regression model. The responder rate was 31.7 %. We found that changes in scores of ADAS-J cog subscales between week 4 and baseline, especially word recognition, can be a good variable to predict subsequent response to galantamine, with approximately 75% of predictive performance. Characteristics of patients, including demographic characteristics, severity of disease and neuropsychological features before treatment were poorly predictive. The present study indicate that initial response to galantamine administration in patients with mild to moderate AD seems to be a reliable predictor of response of consequent galantamine treatment. Patients who show improvement of episodic memory function during the first 4 weeks of galantamine administration may be likely to particularly benefit from galantamine treatment.

## INTRODUCTION

Alzheimer’s disease (AD) is the most common form of dementia, affecting over 25 million people in the world [1]. At present, there are no therapeutic interventions that halt or reverse disease progression, and the currently available medications, typically acetyl cholinesterase inhibitors (AChEIs), are just a palliative therapy for AD symptoms. Various AChEIs have been used in Europe, the United States and Asia, most of which have shown significant clinical efficacy and safety in the treatment of AD. Galantamine hydrobromide (Reminyl^®^) is one of such AChEIs for patients with mild to moderate AD. Galantamine is characterized by two pharmacological mechanisms, inhibition of acetylcholine esterase and allosterically modulating ligand actions by binding to nicotinic acetylcholine receptors [2]. Galantamine maintains acetylcholine concentration in the synaptic cleft by the two mechanisms and thereby compensate the decline of cholinergic function in AD patients.

Although options for pharmacotherapy of AD have been extending, there still has been no consistent position and little supporting evidence on how to choose optimal treatment for patients. In addition, the efficacy of AChEIs is known to differ between patients. Although main efficacy of AChEIs is represented as slowing the cognitive decline caused by AD pathology, some patients treated with AChEIs, so called ‘responder’ show significant improvement of cognitive symptoms. Several studies demonstrated that about one third of patients treated with AChEIs showed such a significant response [3, 4]. Patients with dementia, caregivers, and physicians are concerned with clinically important improvement rather than statistical significances demonstrating clinical trials. Several studies have used a change of 4 points or more on the ADAS-cog scale to define a clinically important improvement for mild to moderate dementia [5]. In this context, the prediction who would be likely to be a responder is a clinically important issue.

The prediction of response to AChEIs has been investigated in various aspects (see Table **3**); 1) prediction based on neuropsychiatric features [6-8], 2) prediction based on genetic features, mostly ApoE4 genotype [9-15], 3) prediction based on the neurochemical features, including CSF biomarkers such as amyloid beta and tau [8, 16], 4) prediction based on neurological features [17-25] and 5) prediction using multivariate including sex, age, ApoE genotype, etc [26]. However, high-accuracy predictors have not been identified thus far, which closely linked to response to AChEIs.

We therefore investigated predictive factors associated with response of galantamine treatment on cognitive symptoms, by a post-hoc analysis of a 24 weeks randomized, double-blind, placebo controlled trial to evaluate the efficacy and the safety of galantamine in the treatment of Japanese patients with mild to moderate AD (A clinical trials in Japan: GAL-JPN-5; ClinicalTrials.gov Identifier:NCT00814801) [27].

## SUBJECTS/MATERIALS AND METHODS

### Subjects

Subjects were mild to moderate AD patients participated in the GAL-JPN-5 (ClinicalTrials.gov Identifier: NCT00814801) [27]. GAL-JPN-5 is a Japan Phase Ⅲ study conducted from January 2006 to September 2008 to assess the effect of galantamine on patients with mild to moderate AD. The study was a multicenter, randomized, double-blind, placebo-controlled trial. Patients initially underwent 4 weeks of observation period, followed by 24 weeks of double-blinded study period. Patients were not eligible to enter the double-blinded study if they received other anti-AD drugs (donepezil, rivastigmine, memantine) during the observation period. Patients were eligible to enter the trial if they met all of the following criteria; 1) A diagnosis of probable AD according to criteria of the National Institute of Neurological and Communicative Disorders and Stroke-Alzheimer’s Disease and Related Disorders Association (NINCDS-ADRDA) [28] with Mini-Mental State Examination (MMSE) [29] scores between 10 and 22, and with ADAS-J cog total scores not less than 18, scores of both orientation and word recall not less than 1, and with Mental Function Impairment Scale (MENFIS) [30] scores of both space and time orientation not less than 1. 2) No coexisting other neurodegenerative disease. Patients were initially randomized in a 1:1:1 ratio to placebo, galantamine 16 mg or 24 mg. During dose escalation, patients in the galantamine group received 4 mg twice daily for weeks 1-4 and 8 mg twice daily for weeks 5-8. During the weeks 9-24, patients in the 16 mg/day group received 8 mg twice daily, and patients in the 24 mg/day group received 12 mg twice daily.

### Datasets

Datasets of the Japanese version of the Alzheimer’s Disease Assessment Scale-cognitive subscale (ADAS-J cog) [31] up to 24 weeks were used for the present analyses and excluded data that were not completed until 24 weeks. The analysis datasets included a group underwent galantamine treatment with a maintenance dose of 16 mg/day (N=153) and a group with that of 24 mg/day (N=150) (totally N=303). 

### Classification of Responders/Non-responders

Among the galantamine-treated groups, patients whose ADAS-J cog scores improved by 4 or more points at 24 weeks of treatment were classified as responders and others were classified as non-responders, according to the definition of “responder” described in NICE guideline [32].

### Screening of Predictive Factors

Based on the definition above, among the total 303 subjects 96 (31.7%) were classified as responders and 207 (68.3%) were classified as non-responders. Data were subsequently divided equally into two parts (2-fold cross validation), one is for modeling (responder: 48, non-responder: 104) and the other is for prediction (responder: 48, non-responder: 103). By random sampling with replacement (bootstrapping), 10 pairs of each dataset were produced. The factors examined are daily dosage of galantamine, demographic factors, scores of MMSE at baseline, scores of ADAS-J cog at baseline, scores of ADAS-J cog at week 4, the difference between at week 4 and baseline of the ADAS-J cog, scores of MMSE subscales at baseline, scores of ADAS-J cog subscales at baseline, scores of ADAS-J cog subscales at week 4 and the difference between them. We analyzed data at week 4 since it could be beneficial for physicians to provide patients with optimal treatment regimen at early stage of AD. Details are described as follows;

1. Daily dosage of galantamine (16mg, 24mg)
2. Demographic factors: sex, age, disease duration
3. Scores of MMSE at baseline, scores of ADAS-J cog at baseline, scores of ADAS-J cog at week 4, and the difference between them.
4. Scores of MMSE subscales at baseline: orientation (year, season, day, month, date, prefecture, city, hospital, floor, district), recall (objects, number), calculation, delayed recall, language (pencil, watch), reading, command (take a paper, fold it in half, put it on the desk), command (write a sentence), writing and drawing
5. Scores of ADAS-J cog subscales at baseline: word recall, language, comprehension of spoken language, word finding difficulty, commands, naming objects and fingers, constructional praxis, ideational praxis, orientation, word recognition task, word recall task (abbreviated as follows; recall, language, comprehension, finding, commands, naming, constructional, ideational, orientation, word recognition, task)
6. Scores of ADAS-J cog subscales at week 4: same as above
7. Difference between week 4 and baseline of the scores of ADAS-J cog subscales: same as above

First of all we calculated effect size of these scores, i.e., we compared difference of the scores of the factors between the responder and the non-responder groups divided by their pooled standard deviation with the use of dataset for modeling, with an underlying assumption that the factor having larger effect size could have higher predictive performance.

### Validation of Predictive Performance

After the screening of the predictive factors described above, a factor with the largest effect size was treated as the most predictive factor. Then a logistic regression analysis was conducted with the responder/non-responder as the binary response variables, and the scores of subscales of the factor as explanatory variables. Firstly, all variables were used for model building as a full model. Secondly, selected variables (significant by Wald’s chi-square, p<0.05) were used for model building as partial models. By maximum likelihood method the regression parameters (coefficients) of the logistic model were determined using the dataset for modeling. Then the model was applied to the other dataset for prediction. These datasets were produced by 1000 times of resampling (bootstrapping). Predictive performance was assessed by receiver operating characteristics (ROC) curve [33]. The endpoints of the predictive performance are the area under the curve (AUC) of ROC, sensitivity and specificity. The sensitivity* and specificity* were determined at the cutoff point where sensitivity-(1-specificity) is maximal in the ROC curve.

### Statistical Analysis

All statistical analyses were conducted with the use of SAS^®^9.2 (SAS Institute Inc., Cary, NC, USA)

## RESULTS

### Responder Rate

Among 303 patients received galantamine, 96 (31.7%) were classified as responders. Among the 96 responders, 44 underwent maintenance dose of 16 mg/day, and 52 underwent that of 24 mg/day. Among the 207 non-responders, 109 underwent maintenance dose of 16 mg/day, and 98 underwent that of 24 mg/day. The association between the two categorical variables (responder/non-responder, 16mg/24mg) was not significant (p=0.326, by chi-square test of independence).

### Effect Size of the Factors Examined

**Fig. (1):**
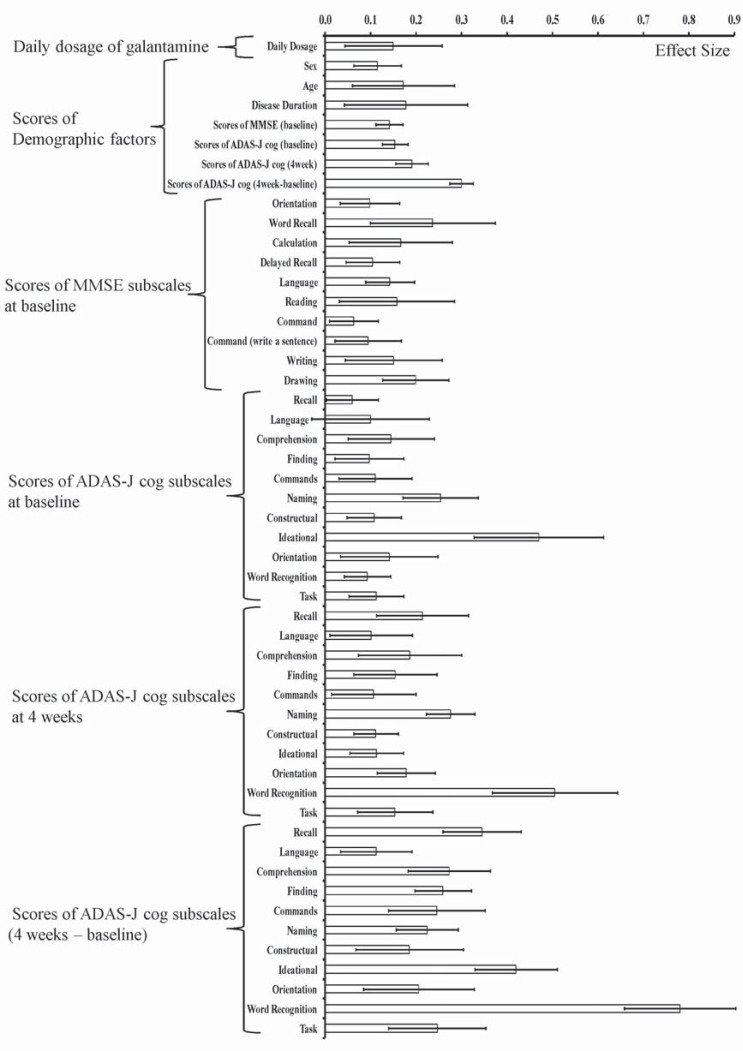
Effect size of each factor and its subscales. Data are mean±standard deviation of 10 bootstrapped datasets.

The effect size of each factor was summarized as shown in (Fig. **1**).

Among the mean values described above, the effect size of the difference between at week 4 and at baseline of the scores of ADAS-J cog subscales was the largest of all factors (nearly 0.3). Among the differences of the ADAS-J cog subscales, the effect size of the score of word recognition was the largest, which was nearly 0.8. It can be concluded that change in the scores of ADAS-J cog subscales at week 4 from baseline was the most predictive factor, thus the subscales were used as input variables for the following logistic regression analysis.

### Predictive Performance

**Fig. (2):**
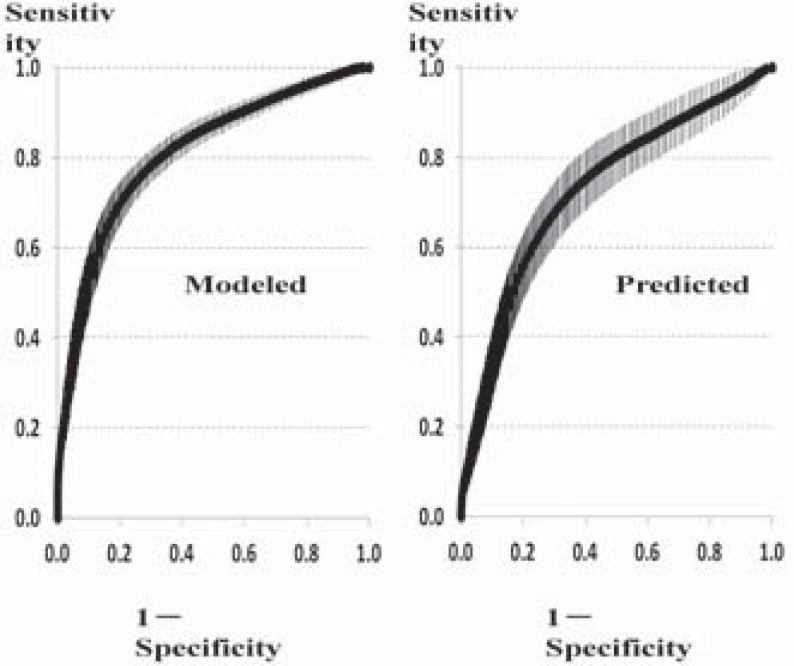
ROC curves using scores of all 11 subscales as input variables (full model). Data are mean±standard deviation of 1000 bootstrapped datasets.

The predictive performance was estimated by ROC curves as shown in (Fig. **2**), using scores of all 11 subscales of the difference between week 4 and baseline of ADAS-J cog as input variables (full model).

The results of AUC of ROC, sensitivity and specificity using the full model were as follows (mean ± standard deviation). 

**Table:** 

AUC of ROC:	0.808±0.029	(modeled),	0.732±0.033
(predicted)
Sensitivity:	0.750±0.074	(modeled),	0.690±0.087
(predicted)
Specificity:	0.789±0.070	(modeled),	0.735±0.084
(predicted)

The regression parameters, Wald’s chi-square, p-values, adjusted odds ratios and their 95% confidence intervals are summarized in (Table **1**). As shown in (Table **1**), the significant variables were ideational, orientation and word recognition. Then we scrutinized these variables into one or two variables to elucidate their predictive performance. As also shown in (Table **2**), the most predictive variable was recognition. The predictive performance, ROC_AUC, sensitivity and specificity of the full model (11 items of ADAS J-cog) and the partial models (Ideational + Orientation + Word recognition, Ideational + Word recognition, Ideational alone, Orientation alone and Word recognition alone) with the use of datasets for prediction were as follows

AUC of ROC: 0.732±0.033 (full model), 0.729±0.033 (Ideational + Orientation + Word recognition,), 0.721±0.032 (Ideational + Word recognition), 0.602±0.035 (Ideational alone), 0.547±0.053 (Orientation alone), 0.710±0.032 (Word recognition alone)

Sensitivity: 0.690±0.087 (full model), 0.650±0.096 (Ideational + Orientation + Word recognition,), 0.709±0.089 (Ideational + Word recognition), 0.521±0.131 (Ideational alone), 0.478±0.275 (Orientation alone), 0.749±0.059 (Word recognition alone) 

Specificity: 0.735±0.084 (full model), 0.749±0.088 (Ideational + Orientation + Word recognition,), 0.683±0.085 (Ideational + Word recognition), 0.640±0.047 (Ideational alone), 0.632±0.277 (Orientation alone), 0.640±0.047 (Word recognition alone).

The order of predictive performance was word recognition > ideational > orientation. The predictive performance by the full model (11 variables), 3 variables, 2 variables and word recognition were almost equivalent (moderate accuracy).

**Fig. (3):**
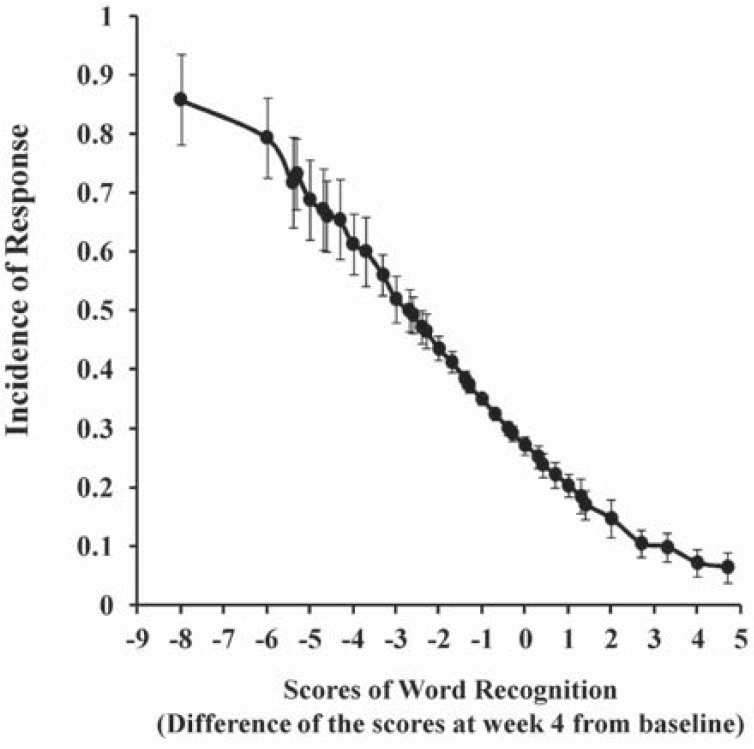
The score of word recognition (difference of the scores of ADAS-J cog subscales) and its relevance to the incidence of response. Data are mean±standard deviation of 20 bootstrapped datasets.

Since among the ADAS-J cog subscales, word recognition was found to be the most predictive subscale when assessed difference of their scores between week 4 and baseline. The score and its relevance to the incidence of response were depicted in the (Fig. **3**), which showed that incidence of response was larger when the score was lower. The mean value of the score was -1.49, indicating that long-term response to galantamine could be predicted when change of the score of word recognition at week 4 from baseline is around 1.5.

## DISCUSSION

In the present study, we retrospectively analyzed data from a randomized, double-blind, placebo controlled trial to evaluate the efficacy and the safety of galantamine in the treatment of 303 patients with mild to moderate AD. We found that response to galantamine treatment, i.e., whether ‘responder’ or ‘non-responder’, can be predictable by the changes of the scores of subscales of the ADAS-J cog, especially, improvement of word recognition during the first 4 weeks of galantamine administration. The result suggests that changes in episodic memory function caused by initial galantamine treatment, even at the titration period, should be a predictor of the response to consequent galantamine treatment. Patients who show improvement of episodic memory function during the first 4 weeks of galantamine administration may be likely to be particularly beneficial to subsequent galantamine treatment. Similar results were reported by Kavanagh *et al.*, where they analyzed six randomized, double-blind, and placebo controlled trials of galantamine treatment and found the relationship between short term (2-5 month) response to galantamine treatment and long term (18 months) benefit of galantamine treatment [34]. Although the evaluated period for both predictor and long-term outcome were different, the result of their study is essentially the same as that of ours, in terms of the fact that initial response to galantamine treatment could be a reliable predictor for long-term outcome of galantamine treatment. A study with Baysian statistics also demonstrated the highly significant correlation between the short-term response and the long-term one [35].

It is noteworthy that dosage, demographic characteristics, and scores of cognitive subscales at baseline were poorly predictive. It suggests that prediction of long-term response to galantamine treatment by using pre-treatment information should be difficult.

Several studies have attempted to find predictors of AChEIs treatment as summarized in (Table **3**). However, no consistently strong factors have emerged thus far [6-26]. For example, ApoE polymorphism is a well known risk factor of getting AD and its disease progression [36]. Furthermore, ApoE4 genotype is associated with lower ChAT levels and a more severe cholinergic deficit, therefore, the presence of the risk allele may predict a poorer response to AChEI therapy [37]. However, the results of studies that assessed whether ApoE genotype was a predictor of response to AChEI therapy were inconsistent [9-15]. One possibility for such varying results is that the studies involve different AChEIs. Although all of them inhibit AChE, other pharmacologic aspects of the compounds differ. Theoretically, such differences may translate to clinical differences, but the similarity of response rates among different AChEIs suggests that the mechanism of the effect should be similar. Indeed, the responder rate of the present study was 31.7 % and was similar to those of previous studies [3, 4]. Another possibility is the methodological differences, including difference in the study design (open-label or blinded), difference in the period of treatment and evaluation, difference in applied cognitive scales (ADAS-cog or MMSE), and the consequent differences of definition of the ‘responder’ amongst the studies could contribute to the discrepancies among the studies.

The present study has two advantages over previous studies. First, the present results were obtained from a double blind, randomized, placebo-controlled trial with a large sample size. Several previous studies of prediction of response to AChEI treatment were done with the data from open label trials with relatively small samples [7, 12, 17, 18, 24]. Second, most of the previous studies did not perform cross-validation of the predictive models, thus the results could be model-specific and labile [38]. Therefore, replication of the results across the studies should be necessary to generalize the results of the studies. On the other hand, we applied a two-fold cross-validation, in which we split the data into two subsets, one for model building and the other for validation. The subsets were randomly sampled with replacement 1000 times (bootstrapping). Such a procedure allows us to generalize the results of the present study. Therefore, we believe that initial improvement of episodic memory function after galantamine administration could be a reliable predictor of preferable long-term outcomes and our predictive model could be applied to other cohorts.

Amongst the possible predictors of improvement in cognitive outcomes, qualitative electroencephalogram profile after a single dose of tacrine was consistently found to be a good predictor of cognitive response. However, 3 out of 4 studies were open label and all the study were conducted with small samples [17, 18, 24]. Prospective trials with second-generation AChEIs should be preferable. Disease progression rate seems to be another reliable predictor. Farlow *et al.* studied the data from a multicentre, double-blind, randomized, placebo-controlled trial of rivastigmine (and open-label extension) to assess the relation between AD progression rate and response to rivastigmine [6]. They found that patients with rapidly progressing symptoms measured by ADAS-cog and Progressive Deterioration Scale were more likely to respond to rivastigmine treatment [6]. Considering the clinical setting in the real world, however, it is difficult to evaluate accurately disease progression rate before start of the treatment. Contrary to disease progression rate, initial improvement of recent memory function measured by the delayed recall test is easy to assess in the clinical setting. 

What are underlying mechanisms of the results of our study? We assume that both initial and long-term response to galantamine administration might be associated with functional state of the cerebral cholinergic system in each patient. Although the functional status of the cholinergic system is thought to contribute significantly to symptoms in AD, the degree of brain pathology in AD does not often have a direct relationship to the degree of clinical symptoms [39]. Recent studies revealed that so called ‘cognitive reserve’ should be associated with such a discrepancy [40]. We consider that severity of disease and neuropsychological features before treatment may be affected by ‘cognitive reserve’ as well as pathology of AD and do not precisely reflect functional state of the cerebral cholinergic system in each AD patient, therefore, severity of disease and/or neuropsychological features is not a good predictor for long-term outcomes of AChEIs treatment. Considering the intimate relationship between episodic memory function and the cerebral cholinergic system, initial changes of recent memory function caused by pharmacological intervention might be one of reliable markers of functional status of the cerebral cholinergic system in each AD patient. A recent pharmacological functional magnetic resonance imaging (ph fMRI) study demonstrated that galantamine challenge affected brain activity during the face recognition task in memory related brain regions in AD and mild cognitive impairment, suggesting that ph fMRI challenge tests should prove to be a valuable instrument to examine the functional status of central neurotransmitter systems in a disease and be helpful to assess neurotransmitter system pathology, to monitor disease progression and to predict response to pharmacological therapy [41]. Since the accuracy of our predictive model was moderate, future studies combining molecular imaging techniques (e.g. Positron Emission Tomography) and ph fMRI in order to relate cholinergic receptor status to signal changes in specific brain structures and corresponding clinical phenotypes (initial response to galantamine treatment) would clarify our speculation and would improve the accuracy of our predictive model.

Finally, several limitation of the present study should be mentioned. Firstly, this is a retrospective analysis of the data from a study with a relatively short term period (24week), therefore prospective studies with a longer treatment period are required to validate our results. However, the timing of decision making for switching one type of ChEIs to another type of ChEI is 6 months after the start of treatment [42]. The present study suggests the possibility of early decision making of strategy of pharmacological treatment of AD. Secondary, some important patient characteristics associated with disease progression and risk of disease, such as ApoE genotype and education level were not collected. However, as mentioned previously, results of studies to investigate relationship between ApoE genotype and response to AChEIs treatment were inconsistent [9-15]. Furthermore, four randomized placebo-controlled trials with large samples agreed that ApoE was not a good predictor of response to AChEIs [9, 11, 14, 15]. We are skeptical about the possibility that further study of ApoE could reveal an important difference. Lower education level is also known to be a well known risk factor of Alzheimer’s disease [43]. *Wattmo et al.* reported an opposite effect of education level on long-term outcome of AD, high education level was a risk factor of faster cognitive decline [26]. Future studies are warranted to clarify how education level affects long-term response to AChEIs treatment.

In conclusion, initial response to galantamine administration in patients with mild to moderate AD seems to be a reliable predictor of subsequent galantamine treatment. Although several issues remain to be clarified, the results of the present study suggest that galantamine have greater treatment effects in patients who show improvement of recent memory function during the first 4 weeks of galantamine administration.

## Figures and Tables

**Table 1.** The regression parameters, Wald‘s chi-square, p values, adjusted odds ratios and their 95% confidence intervals.

Variables	Coefficients	S.E.	Chi-square	p-value	Adjusted OR	Lower 95%CI	Upper 95%CI
Recall	0.382	0.256	2.233	0.135	1.466	0.888	2.421
Language	-0.215	0.459	0.219	0.640	0.807	0.328	1.985
Comprehension	0.392	0.475	0.682	0.409	1.480	0.584	3.755
Finding	0.544	0.415	1.722	0.189	1.724	0.764	3.887
Commands	0.218	0.281	0.602	0.438	1.244	0.717	2.157
Naming	0.270	0.495	0.298	0.585	1.310	0.497	3.457
Constructional	0.351	0.392	0.802	0.370	1.421	0.659	3.066
Ideational	0.296	0.132	5.005	0.025*	1.344	1.037	1.742
Orientation	0.322	0.160	4.053	0.044*	1.380	1.009	1.887
Word Recognition	0.474	0.151	9.887	0.002**	1.606	1.195	2.159
Task	0.560	0.456	1.507	0.220	1.751	0.716	4.282

* p<0.05

** p<0.01

**Table 2.** The regression parameters, Wald’s chi-square, p-values, adjusted odds ratios and their 95% confidence intervals in one or two subscale of ADAS-Jcog

Variables	Coefficients	S.E.	Chi-square	p-value	Adjusted OR	Lower 95%CI	Upper 95%CI
Ideational	0.269	0.118	5.224	0.022*	1.308	1.039	1.647
Orientation	0.287	0.149	3.706	0.054	1.333	0.995	1.785
Word Recognition	0.446	0.114	15.402	0.00009**	1.562	1.250	1.952
**Variables**	**Coefficients**	**S.E.**	**Chi-square**	**p-value**	**Adjusted OR**	**Lower 95%CI**	**Upper 95%CI**
Ideational	0.235	0.113	4.301	0.038*	1.265	1.013	1.581
Word Recognition	0.412	0.108	14.525	0.0001**	1.510	1.221	1.866
**Variables**	**Coefficients**	**S.E.**	**Chi-square**	**p-value**	**Univariate OR**	**Lower 95%CI**	**Upper 95%CI**
Ideational	0.232	0.106	4.765	0.029*	1.261	1.024	1.552
**Variables**	**Coefficients**	**S.E.**	**Chi-square**	**p-value**	**Univariate OR**	**Lower 95%CI**	**Upper 95%CI**
Orientation	0.162	0.135	1.434	0.231	1.176	0.902	1.532
**Variables**	**Coefficients**	**S.E.**	**Chi-square**	**p-value**	**Univariate OR**	**Lower 95%CI**	**Upper 95%CI**
Word Recognition	0.406	0.106	14.683	0.0001**	1.500	1.219	1.846

* p<0.05

** p<0.01

**Table 3.** Predictive variables for response to AchEIs in previous studies

Features	Author/year	AChEIs	Predictors for Response to AChEIs	Responder Definition	Cut-off	Cross Validation	Results of Predictor Performance
Neuropsychiatric	Mega/1999	donepezil	Behavioral responses	NPI at 8 wk	4	-	Predictive(decreased delusion, etc.)
	Farlow/2001	rivastigmine	pretreatment progression rate	ADAS-cog at 52 wk	-	-	Predictive (the rate linked to response)
	Wallin/2009	3 AChEIs	pretreatment progression rate	MMSE at 2, 6 mo	2	-	Predictive (the rate linked to response)
	Sakiyama/2012	galantamine	ADAS-J cog (change from baseline to 4w)	ADAS-J cog at 24 wk	4	+	Predictive (ROC_AUC=0.732)
Genetic	Farlow/1999	metrifonate	ApoE4 genotype, sex	ADAS-cog at 12 or 26 wk	-	-	Not predictive
	Oddoze/2000	donepezil	ApoE4 genotype	MMSE at 6 mo	-	-	Predictive (ApoE4 linked to response)
	Wilcock/2000	galantamine	ApoE4 genotype	ADAS-cog at 3,6 mo	4	-	Not predictive
	Aerssens/2001	galantamine	ApoE4 genotype (pooled)	ADAS-cog after treatment	-	-	Not predictive
	Winblad/2001	donepezil	ApoE4 genotype, sex	Gottfries-Brane-Steen	-	-	Not predictive
	Farlow/2004	rivastigmine	ApoE4 genotype	ADAS-cog after treatment	-	-	Not predictive
	Suh/2006	galantamine	ApoE4 genotype	ADAS-cog, CIBIC-plus, etc.	-	-	Not predictive
Neurochemical	Sobow/2009	rivastigmine	Aβ42 in plasma	ADAS-cog at 6 mo	3	-	Predictive(higher in responders)
	Wallin/2009	3 AChEIs	Aβ42, T-Tau, P-Tau	MMSE at 2, 6 mo	2	-	Not predictive
Neurological	Allhainen/1991	tacrine	EEG alpha-theta ratio	MMSE after 4 wk	-	-	Predictive(higher in responders)
	Allhainen/1993	tacrine	EEG alpha-theta ratio	MMSE after 7 wk	-	-	Predictive(higher in responders)
	Knott/2000	tacrine	EEG alpha frequency	MMSE at 12 wk	-	-	Predictive(higher in responders)
	Almkvist/2001	tacrine	EEG alpha-theta ratio	Based on attention test	-	-	Predictive(higher in responders)
	Brown/2003	donepezil	mAChR binding in insular cortex	MMSE&ADAS-cog at 12 wk	2,4	-	Predictive (lower in responders)
	Connelly/2005	3 AChEIs	medial temporal lobe atrophy	MMSE&DDST at 6 mo	-	-	Predictive (lesser in responders)
	Babiloni/2006	donepezil	EEG cortical rhithmicity	Clinical Information at 12 mo	-	-	Predictive(decreased occipital sources)
	Kanetaka/2008	donepezil	atrophy and perfusion at baseline	MMSE at 14-18 wk	4	-	Predictive (ROC_AUC=0.781)
	Shimada/2011	donepezil	FDG-PET	ADAS-cog baseline	-1	-	Predictive (frontal occipital FDG uptake)
Overall	Wattmo/2011	3 AChEIs	males, aged, ApoE4(-), NSAIDs, etc	MMSE&ADAS-cog at 36 mo	-	-	Predictive

## References

[1] Brookmeyer R, Johnson E, Ziegler-Graham K, Arrighi HM (2007). Forecasting the global burden of alzheimer's disease. Alzheimers Dement.

[2] Nagino K, Shikinami K, Saito T, Harada Y (2011). Pharmacological and
clinical profiles of galantamine (reminyl((r)))]. Nihon Yakurigaku Zasshi.

[3] Giacobini E (2000). Cholinesterase inhibitor therapy stabilizes symptoms of alzheimer disease. Alzheimer Dis Assoc Disord.

[4] Matthews HP, Korbey J, Wilkinson DG, Rowden J (2000). Donepezil in alzheimer's disease: Eighteen month results from southampton memory clinic. Int J Geriatr Psychiatry.

[5] Raina P, Santaguida P, Ismaila A, Patterson C, Cowan D, Levine M (2008). Effectiveness of cholinesterase inhibitors and memantine for treating dementia: evidence review for a clinical practice guideline. Ann Intern Med.

[6] Farlow MR, Hake A, Messina J, Hartman R, Veach J, Anand R (2001). Response of patients with alzheimer disease to rivastigmine treatment is predicted by the rate of disease progression. Arch Neurol.

[7] Mega MS, Masterman DM, O'Connor SM, Barclay TR, Cummings JL (1999). The spectrum of behavioral responses to cholinesterase inhibitor therapy in alzheimer disease. Arch Neurol.

[8] Wallin AK, Hansson O, Blennow K, Londos E, Minthon L (2009). Can csf biomarkers or pre-treatment progression rate predict response to cholinesterase inhibitor treatment in alzheimer's disease?. Int J Geriatr Psychiatry.

[9] Aerssens J, Raeymaekers P, Lilienfeld S, Geerts H, Konings F, Parys W (2001). Apoe genotype: No influence on galantamine treatment efficacy nor on rate of decline in alzheimer's disease. Dement Geriatr Cogn Disord.

[10] Farlow M, Lane R, Kudaravalli S, He Y (2004). Differential qualitative responses to rivastigmine in apoe epsilon 4 carriers and noncarriers. Pharmacogenomics J.

[11] Farlow MR, Cyrus PA, Nadel A, Lahiri DK, Brashear A, Gulanski B (1999). Metrifonate treatment of ad: Influence of apoe genotype. Neurology.

[12] Oddoze C, Michel B, Lucotte G (2000). Apolipoprotein e e4 allele predicts a better response to donepezil therapy in alzheimer's disease. Alzheimer's Report.

[13] Suh GH, Jung HY, Lee CU, Oh BH, Lee SK, Lee N (2006). Effect of the apolipoprotein e epsilon4 allele on the efficacy and tolerability of galantamine in the treatment of alzheimer's disease. Dement Geriatr Cogn Disord.

[14] Wilcock GK, Lilienfeld S, Gaens E (2000). Efficacy and safety of galantamine
in patients with mild to moderate alzheimer's disease: Multicentre
randomised controlled trial. Galantamine international-1
study group. BMJ.

[15] Winblad B, Engedal K, Soininen H, Verhey F, Waldemar G, Wimo A (2001). A 1-year, randomized, placebo-controlled study of donepezil
in patients with mild to moderate ad. Neurology.

[16] Sobow T, Kloszewska I, Flirski M, Liberski P (2009). Predictors of long-term
treatment effect of rivastigmine in alzheimer's disease: A role
for beta-amyloid plasma levels?. Neurol Neurochir Pol.

[17] Alhainen K, Partanen J, Reinikainen K, Laulumaa V, Soininen H, Airaksinen M (1991). Discrimination of tetrahydroaminoacridine responders by a single dose pharmaco-eeg in patients with alzheimer's disease. Neurosci Lett.

[18] Alhainen K, Riekkinen PJ (1993). Discrimination of alzheimer patients responding to cholinesterase inhibitor therapy. Acta Neurol Scand Suppl.

[19] Almkvist O, Jelic V, Amberla K, Hellstrom-Lindahl E, Meurling L, Nordberg A (2001). Responder characteristics to a single oral dose of cholinesterase inhibitor: A double-blind placebo-controlled study with tacrine in alzheimer patients. Dement Geriatr Cogn Disord.

[20] Babiloni C, Cassetta E, Dal Forno G, Del Percio C, Ferreri F, Ferri R (2006). Donepezil effects on sources of cortical rhythms in mild
alzheimer's disease: Responders vs. Non-responders. Neuroimage.

[21] Brown D, Chisholm JA, Owens J, Pimlott S, Patterson J, Wyper D (2003). Acetylcholine muscarinic receptors and response to anti-cholinesterase therapy in patients with alzheimer's disease. Eur J Nucl Med Mol Imaging.

[22] Connelly PJ, Prentice NP, Fowler KG (2005). Predicting the outcome of cholinesterase inhibitor treatment in alzheimer's disease. J Neurol Neurosurg Psychiatry.

[23] Kanetaka H, Hanyu H, Hirao K, Shimizu S, Sato T, Akai T (2008). Prediction of response to donepezil in alzheimer's disease: Combined mri analysis of the substantia innominata and spect measurement of cerebral perfusion. Nucl Med Commun.

[24] Knott V, Mohr E, Mahoney C, Ilivitsky V (2000). Pharmaco-eeg test dose response predicts cholinesterase inhibitor treatment outcome in alzheimer's disease. Methods Find Exp Clin Pharmacol.

[25] Shimada A, Hashimoto H, Kawabe J, Higashiyama S, Kai T, Kataoka K (2011). Evaluation of therapeutic response to donepezil by positron emission tomography. Osaka City Med J.

[26] Wattmo C, Wallin AK, Londos E, Minthon L (2011). Predictors of long-term cognitive outcome in alzheimer's disease. Alzheimers Res Ther.

[27] Homma A, Nakamura Y, Saito T, Shikinami K, Ishida R (2011). A placebo-controlled, double-blind, comparative study of galantamine
hydrobromide in patients with alzheimer-type dementia. Jpn J
Geriatr Psychiatry.

[28] Homma A, Fukuzawa K, Tsukada Y (1992). Development of a japanese version of alzheimer's disease assessment scale (adas). Jpn J Geriatr Psychiatry.

[29] (2009). NICE. Donepezil, galantamine, rivastigmine (review) memantine
for the treatment of alzheimer's disease (amended) includes a review
of nice technology appraisal guidance 19 http://www.Nice.Org.Uk/nicemedia/pdf/ta111guidanceamendedaug09.Pdf. NICE technology appraisal guidance 111 (amended).

[30] Zhou X, Obunchowski N, McClish D (2002). Statistical methods in diagnostic
medicine.

[31] Kavanagh S, Howe I, Brashear HR, Wang D, van Baelen B, Todd M (2011). Long-term response to galantamine in relation to short-term efficacy data: Pooled analysis in patients with mild to moderate alzheimer's disease. Curr Alzheimer Res.

[32] Tanzi RE, Bertram L (2001). New frontiers in alzheimer's disease genetics. Neuron.

[33] Lai MK, Tsang SW, Garcia-Alloza M, Minger SL, Nicoll JA, Esiri MM (2006). Selective effects of the apoe epsilon4 allele on presynaptic cholinergic markers in the neocortex of alzheimer's disease. Neurobiol Dis.

[34] Bishop CM (2006). Pattern recognition and machine learning.

[35] Katzman R, Aronson M, Fuld P, Kawas C, Brown T, Morgenstern H (1989). Development of dementing illnesses in an 80-year-old volunteer cohort. Ann Neurol.

[36] Stern Y (2006). Cognitive reserve and alzheimer disease. Alzheimer Dis Assoc Disord.

[37] Goekoop R, Scheltens P, Barkhof F, Rombouts SA (2006). Cholinergic challenge in alzheimer patients and mild cognitive impairment differentially affects hippocampal activation--a pharmacological fmri study. Brain.

[38] Lindsay J, Laurin D, Verreault R, Hebert R, Helliwell B, Hill GB (2002). Risk factors for alzheimer's disease: A prospective analysis from the canadian study of health and aging. Am J Epidemiol.

[39] McKhann G, Drachman D, Folstein M, Katzman R, Price D, Stadlan EM (1984). Clinical diagnosis of alzheimer's disease: Report of the nincds-adrda work group under the auspices of department of health and human services task force on alzheimer's disease. Neurology.

[40] Folstein MF, Folstein SE, McHugh PR (1975). "Mini-mental state". A
practical method for grading the cognitive state of patients for the
clinician. J Psychiatr Res.

[41] Homma A, Niina R, Ishii T, Hasegawa K (1991). Development of a new rating scale for dementia in the elderly: Mental function impairment scale (menfis). Jpn J Geriatr Psychiatry.

[42] Rota E, Ferrero P, Ursone R, Migliaretti G (2007). Short term response is predictive of long term response to acetylcholinesterase inhibitors in Alzheimer's disease: a starting point to explore Bayesian approximation in clinical practice. Bioinformation.

[43] Segal-Gidan F, Cherry D, Jones R, Williams B, Hewett L, Chodosh J, California Workgroup on Guidelines for Alzheimer's Disease
Management (2011). Alzheimer's disease). management guideline: update
2008. Alzheimers Dement.

